# The Sarcoglycan complex is expressed in the cerebrovascular system and is specifically regulated by astroglial Cx30 channels

**DOI:** 10.3389/fncel.2015.00009

**Published:** 2015-02-02

**Authors:** Anne-Cécile Boulay, Bruno Saubaméa, Salvatore Cisternino, Virginie Mignon, Aurélien Mazeraud, Laurent Jourdren, Corinne Blugeon, Martine Cohen-Salmon

**Affiliations:** ^1^Center for Interdisciplinary Research in Biology (CIRB)/Centre National de la Recherche Scientifique, Collège de France, Unité Mixte de Recherche 7241/Institut National de la Santé et de la Recherche Médicale U1050/Neuroglial Interactions in Cerebral physiopathologyParis, France; ^2^University Pierre et Marie CurieParis, France; ^3^MEMOLIFE Laboratory of Excellence and Paris Science Lettre Research UniversityParis, France; ^4^Faculté de Pharmacie, Variabilité de la réponse aux psychotropes, INSERM UMR-S 1144, Université Paris Descartes, Université Paris DiderotParis, France; ^5^Cellular and Molecular Imaging Facility, Faculté de PharmacieCNRS, UMS 3612, INSERM, US 025, Paris, France; ^6^Institut Pasteur, Human Histopathology and Animal modelsParis, France; ^7^Ecole Normale Supérieure, Institut de Biologie de l’ENS, IBENS, Plateforme GénomiqueInserm, U1024, CNRS, UMR 8197, Paris, France

**Keywords:** astrocyte, Connexin 30, Sarcoglycans, BBB, gap junction

## Abstract

Astrocytes, the most prominent glial cell type in the brain, send specialized processes called endfeet, around blood vessels and express a large molecular repertoire regulating the cerebrovascular system physiology. One of the most striking properties of astrocyte endfeet is their enrichment in gap junction proteins Connexin 43 and 30 (Cx43 and Cx30) allowing in particular for direct intercellular trafficking of ions and small signaling molecules through perivascular astroglial networks. In this study, we addressed the specific role of Cx30 at the gliovascular interface. Using an inactivation mouse model for Cx30 (Cx30^Δ/Δ^; Δ means deleted allele) we showed that absence of Cx30 does not affect blood-brain barrier (BBB) organization and permeability. However, it results in the cerebrovascular fraction, in a strong upregulation of *Sgcg* encoding γ-Sarcoglycan (γ-SG), a member of the Dystrophin-associated protein complex (DAPC) connecting cytoskeleton and the extracellular matrix. The same molecular event occurs in Cx30^T5M/T5M^ mutated mice, where Cx30 channels are closed, demonstrating that *Sgcg* regulation relied on Cx30 channel functions. We further characterized the expression of other Sarcoglycan complex (SGC) molecules in the cerebrovascular system and showed the presence of α-, β-, δ-, γ-, ε- and ζ- SG, as well as Sarcospan. Their expression was however not modified in Cx30^Δ/Δ^. These results suggest that a full SGC might be present in the cerebrovascular system, and that expression of one of its member, γ-SG, depends on Cx30 channels. As described in skeletal muscles, the SGC may contribute to membrane stabilization and signal transduction in the cerebrovascular system, which may therefore be regulated by Cx30 channel-mediated functions.

## Introduction

Astrocytes, the major glial cells in the brain, represent a rather unique example of multifunctional cells in the central nervous system (CNS), being in tight vicinity with neurons and blood vessels, and regulating both neuronal and vascular functions. In particular, astrocytes make intimate contacts with the cerebrovascular system elaborating the so-called gliovascular unit, sheathing blood vessels with specialized processes termed endfeet (Mathiisen et al., 2010). Astrocytes perform a range of vascular regulatory activities, coordinating blood flow with neuronal activity (Petzold and Murthy, 2011) and regulating metabolite transfer to neurons (Bélanger et al., 2011). They also contribute to the blood-brain barrier (BBB) that creates a specific microenvironment vital to the CNS functions, regulating its integrity (Abbott et al., 2006) and its immune quiescence (Alvarez et al., 2011; Argaw et al., 2012; Urich et al., 2012; Jensen et al., 2013). A typical feature of astrocytes is their high level of Connexin (Cx) expression, with two major subunits, Cx43 and Cx30. Cxs assemble by 6 in connexons, which when inserted in the plasma membrane, dock together to form gap junction channels allowing direct astrocyte-to-astrocyte exchange of ions and small signaling molecules up to 1–1.2 kDa. Connexons can also stay in a hemichannel (Hc) conformation and mediate the direct exchange of molecules between the intra- and extracellular milieu (Chever et al., 2014a; Sáez and Leybaert, 2014). Finally, Cxs mediate channel-independent functions, such as cell-cell adhesion and signalization (Elias et al., 2007, 2010; Zhou and Jiang, 2014). To date, Cx30 and Cx43 have been shown to contribute both or individually to several aspects of the brain physiology, such as energy metabolite trafficking (Rouach et al., 2008), neurogenesis (Kunze et al., 2009), myelin maintenance (Lutz et al., 2009; May et al., 2013) and control of synaptic activity (Theis et al., 2003; Dallérac et al., 2013; Chever et al., 2014b; Pannasch et al., 2014).

Interestingly, astroglial Cxs are highly concentrated in perivascular endfeet where they form large gap junction plaques (Simard et al., 2003; Ezan et al., 2012), allowing the elaboration of electrical and chemical astroglial networks around the brain endothelium (Rouach et al., 2008). This molecular and structural organization led us to hypothesize that astroglial Cxs may participate to the regulation of vascular functions. Accordingly, deletion of both Cx43 and Cx30, which uncouples astrocytes (Rouach et al., 2008), has been shown to weaken BBB integrity (Ezan et al., 2012). Here, we addressed the specific contribution of astroglial Cx30 to the BBB organization.

## Materials and methods

### Mice

Animals were kept in pathogen free conditions. Mice of either sex were used in this study except for the transcriptome analysis and *in situ* perfusion experiments for which only males were selected. Cx30^Δ/Δ^ (Δ means deleted allele) are ubiquitously deleted for Cx30 (Boulay et al., 2013). Cx30^T5M/T5M^ mice carry a point-mutated form of Cx30 in place of Cx30, resulting in the replacement of a threonine at position 5 by a methionine (Schütz et al., 2010). Cx30^T5M/T5M^ mice were maintained on a pure C57BL6 genetic background. Cx30^Δ/Δ^ and Cx30^+/+^ (wild-type (WT) mice) had a C57BL6/Balbc genetic background for the transcriptome study and were pure C57BL6 for other experiments.

### Study approval

Experiments and techniques reported here complied with the ethical rules of the French agency for animal experimentation and with the IMTCE (Institut Médicament Toxicologie Chimie Environnement) animal ethics committee (Université Paris Descartes) (agreement number 86-23).

### Isolation of brain vessels, RNA preparation, cDNA libraries and RNA sequencing

Brain vessels from 3-month-old Cx30^Δ/Δ^ mice (Boulay et al., 2013) and control Cx30^+/+^ mice were isolated from whole brains as previously described (Yousif et al., 2007). mRNAs were subsequently purified and sequenced. Purified vessels from 3 mice of each group were pooled. Total RNA was extracted using the Rneasy Lipid tissue kit (Qiagen). Messenger (polyA^+^) RNAs were purified from 1 µg of total RNA using oligo (dT). Libraries were prepared using the strand non-specific RNA-Seq library preparation TruSeq RNA Sample Prep Kits v2 (Illumina). Libraries were multiplexed on one single flowcell lane and subjected to 50 base pair (bp) single read sequencing on a HiSeq 2000 device. A mean of 56 ± 27 million passing illumina quality filter reads was obtained for each samples.

The whole RNA-Seq data analysis was done using the Eoulsan software version 1.1.6 (Jourdren et al., 2012) with the following parameters. Before mapping, polyN read tails were trimmed, reads ≤11 bases were removed, and reads with quality mean ≤12 were discarded. Reads were then aligned against the Mus musculus genome (mm10 genome assembly from UCSC) using the Bowtie mapper (version 0.12.7) (Langmead et al., 2009) using the—best and –k 2 parameters. Alignments from reads matching more than once on the reference genome were removed. To compute gene expression, *Mus musculus* GFF3 genome annotation from UCSC (mm10) was used. All overlapping regions between alignments and referenced exons were counted. Data normalization and differential analysis was performed using the DESeq package version 1.6.10 (Anders and Huber, 2010). RNA sequencing data are available at: http://www.ncbi.nlm.nih.gov/geo/query/acc.cgi?token=qtyrsakstzerxmv&acc=GSE59148.

### Reverse transcription polymerase chain reaction (RT-PCR) and quantitative polymerase chain reaction (qPCR)

Reverse transcription was performed from RNA extracted from purified brain vessels (300 ng) or dissected hippocampus and cortex (1 µg) using the Rneasy kit (Qiagen) and Supercripts II (Life Technologies). Polymerase chain reaction (PCR) was performed on 1 µL of RT reaction using Taq polymerase (Qiagen) and the following primers: Sgcg (γ-SG) forward 5′-TCACCGAGGGCACTCACATA-3′; Sgcg reverse 5′-CCAACCACAACGTCCTGCT3′; Sgca (α-SG) forward 5′-GCCGAGTCCCTCTTCCTATT-3′; Sgca reverse 5′-CCAGAGACACATTGCACCAG-3′; Sgcb (β-SG) forward 5′-AGCATGGAGTTCCACGAGAG-3′; Sgcb reverse 5′-GCTGGTGATGGAGGTCTTGT-3′; Sgcd (δ-SG) forward GTCAGAGCAGACCCCTTCAA ; Sgcd reverse 5′-GATCCACGAGGCAGTCTAGC-3′; Sgce (ε-SG) forward 5′-TCCATCACAGCTCGATTCAG-3′; Sgce reverse 5′-TCTGAGTCTGGTGTGGCAAG-3′; Sgcz (ζ-SG) forward 5′-GAAAGAAATTCATTCCCGAAAGG-3′; Sgcz reverse 5′-GAATCAGGAAAGGTGAAGGCCAA-3′; Sarcospan forward 5′-AGAGGACTTGCTGCTCTTGC-3′; Sarcospan reverse 5′-CCTTTCGGTGTTCACCAAGT-3′. Sgcg qPCR was conducted using SYBR Green PCR master kit (Applied Biosystems). qPCR cycling conditions were 50°C for 2 min, 95°C 10 min, and 40 cycles of 95°C for 15 s and 60°C for 1 min. All experiments were performed in triplicate on an LC480 Roche Light cycler. The relative abundance of amplified cDNA was calculated as 2^−ΔCt^, where ΔCt (change in cycle threshold) equals Ct in Cx30^Δ/Δ^ minus Ct in Cx30^+/+^. Results are expressed as means of (2^−ΔCt^ tested cDNA)/(2^−ΔCt^ RNA18s values). RNA18s forward 5′-TTGAAAATCCGGGGGAGAG-3′; RNA18s reverse 5′-ACATTGTTCCAACATGCCAG-3′. RT-PCR cycling conditions were 95°C for 3 min, and 35 cycles of 95°C for 30 s, 60°C for 30 s and 72°C for 1 min. Negative controls for RT-PCR and qPCR were done on RT reactions performed without reverse transcriptase.

### *In situ* brain perfusion

Mice were anesthetized with ketamine-xylazine (140-8 mg/kg, i.p.) and a polyethylene catheter was inserted into the carotids. The heart was cut and the perfusion was started immediately (flow rate: 2.5 mL/min) to obtain a complete substitution of the blood by the artificial perfusion fluid, a Krebs bicarbonate-buffered physiological saline (mM)(128 NaCl, 24 NaHCO_3_, 4.2 KCl, 2.4 NaH_2_PO_4_, 1.5 CaCl_2_, 0.9 MgCl_2_, 9 D-glucose) containing also [^14^C]-sucrose (0.3 µCi/mL) (Perkin Elmer Life Sciences, Courtaboeuf, France) as a vascular and integrity marker, gassed with 95% O_2_/5% CO_2_ for pH control (7.4) and warmed to 37°C. Sucrose (glucose-fructose) is a low molecular weight disaccharide compound. Being hydrophilic, it does not bind to plasma proteins. Moreover, in contrast to the very specific monosaccharide (e.g., glucose) transporters, sucrose has no dedicated transporter in mammals and thus exhibits negligible BBB/cellular crossing (Takasato et al., 1984). Perfusion was terminated after 120 s by decapitating the mouse. The whole brain was removed from the skull and dissected out on a freezer pack. Tissue and two aliquots of perfusion fluid were placed in tared vials and weighed, digested with Solvable® (Perkin Elmer) and mixed with Ultima gold XR® (Perkin Elmer) for ^14^C counting (Tri-Carb®, Perkin Elmer). In some experiments, human serum albumin (40 g/L) (Vialebex, Paris, France) was added in the perfusion fluid to increase its viscosity and then the hydrostatic pressure (~180 mmHg) according to the Poiseuille’s law (Ezan et al., 2012). The brain vascular volume (V_v_) (µL/g) was calculated using the distribution of [^14^C]-sucrose: V_v_ = X_v_/C_v_ where X_v_ (dpm/g) is the [^14^C]-sucrose measured in the right hemisphere and C_v_ (dpm/µL) is the concentration of [^14^C]-sucrose in the perfusion fluid (Dagenais et al., 2000).

### Immunofluorescence on purified brain vessels

Purified brain vessels prepared as described by (Yousif et al., 2007) were fixed by immersion in PBS/PFA 4% for 20 min at room temperature, rinsed three times in PBS, and immersed in the blocking solution (PBS/NGS 5%/Triton 0.25%) for 1 h at room temperature. They were then incubated with monoclonal anti Gfap (clone GA5, Sigma) (dilution: 1/500), SMA (clone 1A4, Sigma) (dilution: 1/500) in the blocking solution 12 h a 4°C, rinsed three times in PBS, incubated 2 h at room temperature with secondary antibodies Alexa-conjugated goat anti-mouse IgG (Life technology; A11029/A21424), rinsed three times in PBS, and mounted on glass slides in FluormountG for fluorescent revelation. Fluorescence was imaged on a SP5 confocal microscope (Leica).

### Western blot

Purified brain vessels or whole cortex and hippocampus were homogenized in PBS containing 2% SDS and 1x EDTA-free Complete Protease Inhibitor (Roche), sonicated three times at 10 Hz (Vibra cell VCX130) and centrifuged 20 min at 10000 g at 4°C. Supernatants were boiled in 5x Laemmli loading buffer. Protein content was measured using the Pierce 660 nm protein assay reagent (Thermo scientific). Equal amounts of proteins were separated by denaturing electrophoresis in 4–12% or 3–8% (for anti-ZO-1) NuPAGE gradient gel (Invitrogen) and electrotransfered to nitrocellulose membranes. Membranes were analyzed using the following primary antibodies: rabbit anti-Occludin (Invitrogen) (1:500); mouse anti-ZO-1 (Invitrogen) (1:500); mouse anti-Cx43 (BD Biosciences) (1:500); rabbit anti-Aquaporin-4 (Aqp4) (Sigma, St Louis, MO, USA) (1:500); rabbit anti-Claudin-5 (Invitrogen) (1:500); mouse anti-β-Dystroglycan (β-DG) (Novocastra, Newcastle, UK) (1:200); monoclonal anti γ-Sarcoglycan (γ-SG) (Leica) (1:100); horseradish-peroxidase-conjugated (HRP) anti-GAPDH (1:5000); mouse anti-tubulin (Sigma) (1:2000). Secondary antibodies used were HRP-conjugated goat anti-mouse and anti-rabbit antibodies (Amersham) (dilution 1:2000). HRP activity was visualized by enhanced chemiluminescence (ECL) using Western Lightning plus enhanced chemoluminescence system (Perkin Elmer). Chemoluminescence imaging was performed on a LAS4000 (Fujifilm). Tubulin or GAPDH expression was used as a loading reference.

### Electron microscopy

Mice were anesthetized with Ketamine-Xylazine (140-8 mg/kg, i.p.) and transcardially perfused with the fixative (2% Paraformaldehyde, 3% Glutaraldehyde, 3 mM CaCl_2_ in 0.1 M Cacodylate Buffer pH 7.4) for 2 min at 10 mL/min and then for 30 min at 2 mL/min. Brains were removed and left overnight at 4°C in the same fixative. Brain fragments (0.3 × 1 × 1 mm^3^) were then postfixed first in 0.1 M Cacodylate Buffer pH 7.4 + 1% O_s_O_4_ for 1 h at 4°C and then in 1% aqueous Uranyl Acetate for 2 h at RT. After dehydration in graded Ethanol, followed by Propylene Oxyde, the fragments were embedded in Epon. Ultrathin (80 nm) sections were prepared, stained in Lead Citrate and photographed in a Jeol 100S transmission electron microscope (Jeol, Croissy-sur-Seine, France) equipped with a 2k × 2k Orius 830 CCD camera (Roper Scientific, Evry, France).

## Results

### Absence of Cx30 does not perturb BBB integrity

**Figure 1:**
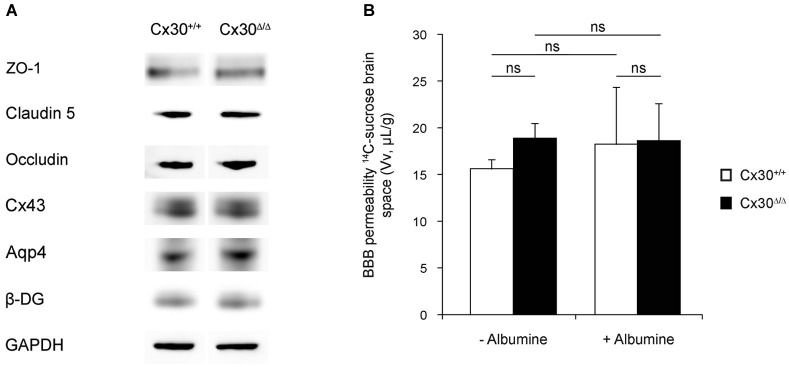
**Astroglial Cx30 is not required for BBB integrity. (A)** Western-blot of tight junction (TJ) proteins ZO-1, Claudin-5 and Occludin, Connexin43, Aquaporin-4 (Aqp4) and β-Dystroglycan (β-DG) in 3-month-old Cx30^+/+^ and Cx30^Δ/Δ^ cortex and hippocampus. GAPDH was used as the loading control (*n* = 4). **(B)** BBB integrity in 3-month-old Cx30^+/+^ and Cx30^Δ/Δ^ mice was assessed by measuring the brain vascular volume (Vv in µL/g) by *in situ* brain perfusion of [^14^C]-sucrose in normal intravascular pressure (−Albumin; 120 mmHg) or increased shear stress and hydrostatic vascular pressure (+Albumin, 180 mmHg). No Albumin: Cx30^+/+^, 15.6 ± 0.5 and Cx30^Δ/Δ^, 18.8 ± 0.4 (*n* = 7); With Albumin: Cx30^+/+^, 18.2 ± 2.2 and Cx30^Δ/Δ^, 18.6 ± 1.2 (*n* = 7). Data are means ± SEM. Kruskal-Wallis test followed by a Dunn’s multiple comparisons test, *p* = 0.2 (ns).

**Figure 2:**
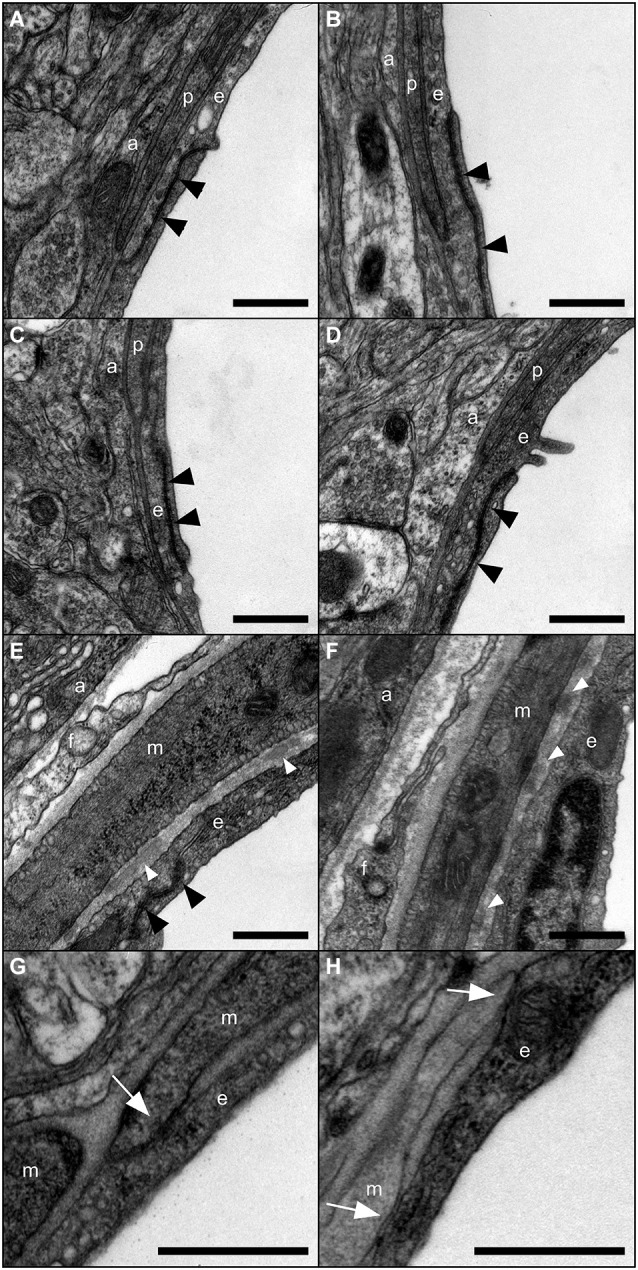
**Ultrastructure of the gliovascular unit in absence of Cx30**. Representative images of the gliovascular unit ultrastructure in 3-month-old Cx30^+/+^ **(A,C,E,G)** and Cx30^Δ/Δ^ **(B,D,F,H)** mice (*n* = 4) within a post-capillary venule **(A,B)** a capillary **(C,D)** and an arteriole **(E,F,G,H)** showing astroglial perivascular endfeet (a), endothelial cells (e) with TJs (black arrowheads), vascular smooth muscle cells (VSMCs) (m) fibrocytes (f) and pericytes (p). Fragments of amorphous elastic material in basal lamina (white arrowheads) and junctional contacts between endothelial and smooth muscle cells (white arrows) are found in arterioles. Scale bars 500 nm.

In a recent study, we demonstrated that deletion of Cx43 and Cx30, the two major astroglial Cxs, modifies endfeet architecture and weakens BBB (Ezan et al., 2012). Here, we addressed the specific contribution of Cx30 to BBB integrity in 3-month-old Cx30-deleted mice (Cx30^Δ/Δ^) where Cx30 is ubiquitously deleted (Boulay et al., 2013) compared to WT (Cx30^+/+^) mice. Western-blot detection of endothelial tight junction (TJ) proteins ZO-1, Claudin-5 and Occludin in 3-month-old Cx30^Δ/Δ^ and Cx30^+/+^ cortex and hippocampus revealed no difference (Figure 1A). The same observation could be done also for Cx43, as well as Aqp4 and β-DG, two molecules decreased in the brain of mice deleted for both Cx30 and astroglial Cx43 (Ezan et al., 2012; Figure 1A). We next measured the brain Vv of Cx30^+/+^ and Cx30^Δ/Δ^ by *in situ* brain perfusion of [^14^C]-sucrose (Dagenais et al., 2000; Ezan et al., 2012; Figure 1B). In this experiment, [^14^C]-sucrose was used as a marker of the vascular space and BBB integrity because it does not cross the BBB significantly during short exposure (Takasato et al., 1984). The distribution of [^14^C]-sucrose Vv was measured in the whole brain after 120 s of *in situ* brain perfusion of regular Krebs at 2.5 mL/min. No statistical difference was noticed between Cx30^+/+^ and Cx30^Δ/Δ^ mice, indicating that BBB integrity was unchanged in absence of Cx30. We next examined the mechanical resistance of the BBB performing *in situ* brain perfusion in presence of human serum albumin in the saline perfusion to increase shear stress and hydrostatic vascular pressure (from 110 to 180 mmHg) (Ezan et al., 2012; Figure 1B). However, even in these conditions, the Vv in Cx30^Δ/Δ^ remained comparable to Cx30^+/+^ and to the Vv recorded without albumin (Figure 1B). Thus albumin-mediated shear stress and vascular pressure increase had no effect on the BBB permeability, indicating no alteration of BBB integrity in absence of Cx30. We finally addressed the ultrastructure of the gliovascular unit in each segment of the vasculature (proximal and distal arterioles, capillaries and veins) in 3-month-old Cx30^Δ/Δ^ compared to Cx30^+/+^ mice (Figure 2). No significant difference was found between Cx30^Δ/Δ^ and Cx30^+/+^ at the ultrastructural level: endothelial TJ were well formed and extended from the vessel lumen to the basal lamina (BL) with no apparent discontinuity, no accumulation of pinocytic vesicles was observed; BL around capillaries was thin and regular except in arterioles where it was occasionally enlarged by fragments of amorphous elastic material; Astroglial perivascular endfeet were in close contact with the BL and showed thin processes with a dense intracellular content and no sign of swelling. In arterioles, vascular smooth muscle cells (VSMCs) were normal and separated from the astroglial endfeet by a regular BL, where macrophages and fibrocytes were found. They showed frequent junctional contacts with endothelial cells. Altogether, these results demonstrate that neither the permeability of the BBB to plasmatic compounds, nor the structure of the gliovascular unit, were perturbed following the genetic inactivation of Cx30.

### Impairment of Cx30 channels upregulates the expression of γ-Sarcoglycan in brain vessels

**Figure 3:**
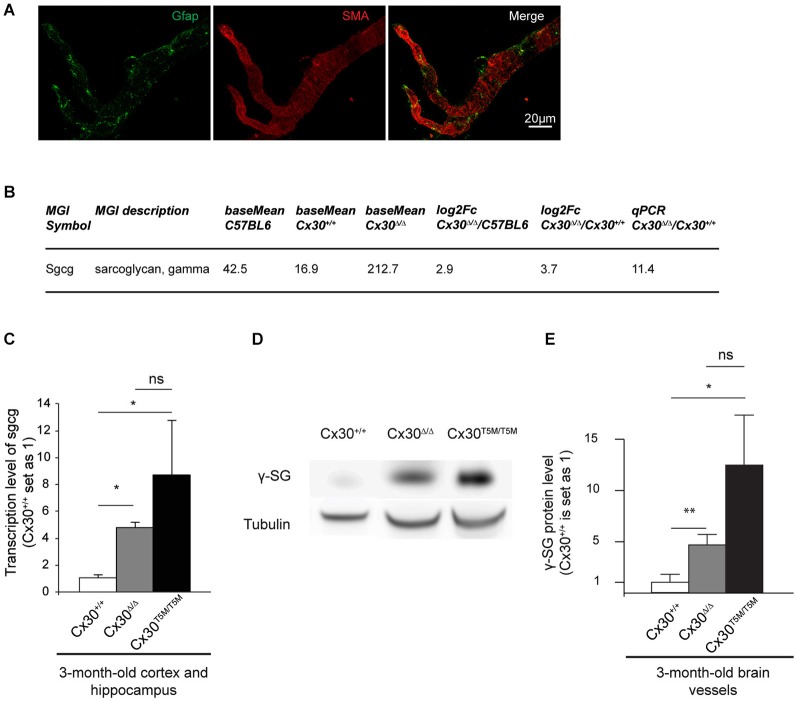
**Impairment of Cx30 channels upregulates the expression of γ-Sarcoglycan in brain vessels. (A)** Characterization of purified brain vessels by Gfap and SMA (Smooth Muscle alpha-Actin) immunostaining. **(B)** Upregulation of *Sgcg* in brain vessels purified from 3-month-old Cx30^Δ/Δ^ compared to Cx30^+/+^ and C57BL6 control mice revealed by RNA sequencing. Base mean indicates the number of reads, Fc indicates Fold change. On the right, Validation of the RNAseq result by qPCR on RNAs purified from brain vessels of Cx30^Δ/Δ^ relative to Cx30^+/+^ control mice. Cx30^+/+^ value is set as 1. **(C)** qPCR analysis on 3-month-old Cx30^+/+^ compared to Cx30^Δ/Δ^ and Cx30^T5M/T5M^ cortex and hippocampus. Cx30^Δ/Δ^, 5.0 ± 0.6 *n* = 4; Cx30^T5M/T5M^, 8.6 ± 1.4 *n* = 4; Cx30^+/+^, 1.0 ± 0.1 *n* = 3. Data are presented as means ± SEM. Mann-Whitney two-tailed test. Cx30^+/+^ vs. Cx30^Δ/Δ^ or Cx30^T5M/T5M^, *p = 0.05; Cx30^Δ/Δ^ vs. Cx30^T5M/T5M^ ns, *p* = 0.5. **(D)** Western-blot of γ-SG (30 kDa) in 3-month-old Cx30^+/+^, Cx30^Δ/Δ^ and Cx30^T5M/T5M^ purified brain vessels. Tubulin (50 kDa) was used as the loading control. **(E)** Western blot quantifications. Cx30^Δ/Δ^, 4.7 ± 1 *n* = 5; Cx30^T5M/T5M^, 12.5 ± 4.9 *n* = 3; Cx30^+/+^, 1.0 ± 0.4 *n* = 6. Data are presented as means ± SEM. Mann-Whitney two-tailed test. Cx30^+/+^ vs. Cx30^Δ/Δ^, **p = 0.009; Cx30^+/+^ vs. Cx30^T5M/T5M^, *p = 0.02; Cx30^Δ/Δ^ vs. Cx30^T5M/T5M^ ns *p* = 0.07.

To further address the role of Cx30 at the cerebrovascular level, we analyzed the transcriptome of isolated brain vessels purified from 3-month-old Cx30^Δ/Δ^ and control mice (Figure 3). As previously described, this preparation contains endothelial cells, VSMCs and pericytes (Yousif et al., 2007). We further verified the absence of astrocytes in our vessel preparation performing Gfap immunostaining on isolated brain vessels. Only few remaining Gfap positive fibers could be detected at the vascular surface but no copurified astrocyte cell bodies (Figure 3A).^1^ Since Cx30^Δ/Δ^ mice were maintained on a mixed C57BL6/Balbc genetic background, and background strongly impacts gene transcription (Iacobas et al., 2012), two distinct WT RNAs libraries, from C57BL6 and C57BL6/Balbc (Cx30^+/+^) mice, were prepared. We then compared the Cx30^Δ/Δ^ and WT transcriptomes and selected changes observed in Cx30^Δ/Δ^ brain vessels whatever the genetic background. Genes with less than 50 reads and/or with fold change less than 2 were not considered. Selected transcriptional variations were further validated by qPCR on independent brain vessel preparations. Consistently with the above data (Figure 1), transcription of junctional molecules as well as pathways implicated in BBB integrity were not modified in absence of Cx30. Among the transcriptional events detected in the Cx30^Δ/Δ^ brain vascular transcriptome, we confirmed the strong upregulation of *Sgcg* encoding γ-SG, a member of the dystrophin-glycoprotein complex (DGC) connecting cytoskeleton and the extracellular matrix (Ozawa et al., 2005; Figure 3C). We next aimed to establish which Cx30 function was involved in the regulation of *Sgcg*. Indeed, Cx30 displays gap junction and Hc functions as well as channel-independent functions (Qu et al., 2009; Pannasch et al., 2014). We confirmed *Sgcg* upregulation in whole cortex and hippocampus and analyzed its transcription level in Cx30^T5M/T5M^ mice, in which the replacement of a threonine by a methionine at position 5 of Cx30 leads to a defective Cx30 channel pore but intact membrane targeting (Schütz et al., 2010; Figure 3C). As observed in Cx30^Δ/Δ^, *Sgcg* was strongly upregulated in Cx30^T5M/T5M^ compared to Cx30^+/+^. We finally tested if the transcriptional upregulation of *Sgcg* led to an increase in γ-SG protein performing a Western-blot of proteins extracted from brain vessels (Figures 3D,E). Consistently with qPCR results, γ-SG was increased in Cx30^Δ/Δ^ and Cx30^T5M/T5M^ brain vessels. These results thus suggest that absence of Cx30 channels leads to the increase of γ-SG in the brain vessels and that* Sgcg* transcription is regulated by astroglial Cx30 channel functions.

### The Sarcoglycan complex is expressed in the brain vessels

**Figure 4:**
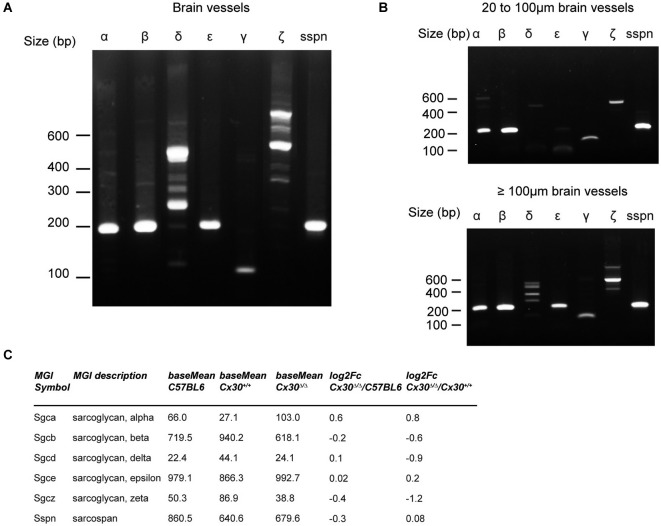
**Composition of the SG complex in brain vessels. (A)** α, β, δ, ε, γ and ζ-Sarcoglycan (SG) as well as Sarcospan were all amplified by RT-PCR on brain vessels purified from 3-month-old control mice. **(B)** RT-PCR analysis of the SG complex in brain vessels retained on a 100 µm filter (>100 µm in diameter) or on a 20 µm filter (from 20 to 100 µm in diameter), base pair (bp). **(C)** RNAseq analysis of α, β, δ, ε, γ and ζ-SG and Sarcospan in brain vessels purified from 3-month-old Cx30^Δ/Δ^ compared to Cx30^+/+^ and C57BL6 control mice. Base mean indicates the number of reads, Fc indicates Fold change.

The Sarcoglycan complex (SGC) is a multimeric transmembrane protein assembly, which has been extensively studied in striated muscle cells (Ozawa et al., 2005). It interacts with the DG and is part of the Dystrophin-associated protein complex (DAPC), bridging the extracellular matrix to the actin cytoskeleton and ensuring membrane stability and force transduction during muscle contraction (Constantin, 2014). In the brain, SG units have been found in the membranes and the cytoplasm of some large cortical neurons as well as in some astrocyte cell bodies (Anastasi et al., 2012). However, the presence of a SGC in the cerebrovascular system had never been documented. Following the finding of a brain vascular γ-SG, we tested if other units of the SGC were expressed performing RT-PCR experiments in purified brain vessels. As shown in Figure 4A, transcripts encoding all known SG units, α, β, δ, ε, γ and ζ as well as Sarcospan were amplified. δ- and ζ-SG amplification showed additional higher bands, which could correspond to uncharacterized transcript isoforms (Figure 4A). We next determined if SGC molecules were expressed in all vessel types by repeating this RT-PCR study on brain vessels separated by size (Yousif et al., 2007; Figure 4B). Interestingly, δ- and ε-SG were expressed only in vessels with a diameter >100 µm (Figure 4B). Finally, our transcriptome analysis showed that α, β, δ, ε, γ and ζ-SG and Sarcospan expression was not changed in Cx30^Δ/Δ^ purified vessels (Figure 4C). Altogether, our results suggest that the whole SGC might be present in brain vessels, with δ- and ε-SG being specific to the largest vessels.

## Discussion

Here, we addressed the role of Cx30, a gap junction protein highly enriched in the astrocyte perivascular endfeet, in the brain vascular physiology. In a previous study, we demonstrated that deletion of Cx30 and Cx43 (DKO mice), the major astroglial Cxs, led to astrocyte endfeet swelling and to a reduced BBB resistance to shear stress (Ezan et al., 2012). None of these effects were observed in Cx30^Δ/Δ^ mice. In addition, in agreement with the absence of BBB leakage, gene expression of junctional molecules and pathways implicated in BBB integrity were not modified in the cerebrovascular fraction in absence of Cx30. In particular, expression of β-DG and Aqp4, two major proteins of the DAPC at gliovascular interface, which were decreased in DKO mice, was not modified in Cx30^Δ/Δ^. Thus, astroglial Cx43, whose expression was unchanged in Cx30^Δ/Δ^ mice, would be sufficient to maintain the BBB integrity and the gliovascular unit structure. However, *Sgcg* encoding γ-SG was highly upregulated in Cx30^Δ/Δ^ mice. Thus, Cx43 is unable to compensate for this upregulation, and Cx30 plays a specific role in the *Sgcg* trancriptional control, which remains to be defined. Recent data have unraveled an unexpected complexity and variety in the Cx functions (Zhou and Jiang, 2014). Independently of their channel functions (GJ and Hc), Cxs have been shown to display adhesive (Elias et al., 2007, 2010), as well as signaling functions (Scemes, 2008). Moreover, Cx30 interacts with actin, tubulin (Qu et al., 2009) and was recently shown to control astroglial morphology through channel-independent functions (Pannasch et al., 2014). Here, *Sgcg* upregulation was also found in Cx30^T5M/T5M^ mice, where only Cx30 channel functions are disrupted (Schütz et al., 2010). These results thus suggest that the Cx30-mediated control of *Sgcg* transcription relies specifically on its channel functions, either gap junction, Hc or both.

γ-SG belongs to the SGC and interacts with β-DG within the muscular DAPC, a transmembrane heteromeric complex linking the cytoskeleton to the BL, crucial to the mechanical signal transduction (Ozawa et al., 2005). In addition to its bridging properties between the extracellular matrix and the cytoskeleton, the DAPC has also been proposed to constitute a putative cellular signaling complex by conferring the scaffold for numerous signaling proteins and ion channels. Along this line, it would influence calcium homeostasis and signaling (reviewed by Constantin, 2014). For instance, the cysteine-rich domain of Dystrophin may represent a functional Calmodulin-binding domain modulating the recruitment of other Dystrophin-associated proteins in a calcium-dependent manner. The DAPC-calcium homeostasis regulation is also mediated by the SGC. For instance, *Sgcg* deletion in mice has been shown to induce the upregulation of Sorcin, a calcium-handling protein, leading to disruption of the DAPC in muscles (Goldstein et al., 2014). In the brain, DAPCs of various compositions have been described at the level of specialized cellular contacts with the extracellular matrix, in GABAergic synapses (Waite et al., 2012), in the choroid plexus (Haenggi and Fritschy, 2006) and at the BBB level in endothelial cells (Zaccaria et al., 2001; del Zoppo and Milner, 2006; Haenggi and Fritschy, 2006), astrocyte endfeet (Haenggi and Fritschy, 2006; Wolburg et al., 2009) and VSMCs (North et al., 1993; Yousif et al., 2013). At the astrocyte endfeet level, the DAPC was shown to anchor the water channel Aqp4, which is critical to regulate ATP release and astrocyte-cell volume, via Ca^2+^-dependent signalizations (Benfenati et al., 2011; Thrane et al., 2011). Based on our RT-PCR study, we demonstrated that all SG units as well as Sarcopan were present at the vascular level in the brain, with a differential expression of δ- and ε-SG in vessels with a diameter >100 µm. Unfortunately, available antibodies against SGC proteins gave very weak signal on sections as well as on isolated brain vessels precluding us to determine their precise cellular and subcellular site of expression. Nevertheless, recent transcriptomic studies showed that none of the SGC molecules are expressed in astrocytes and endothelial cells, except for *Sgcb* found moderately transcribed in the endothelial cells and *Sgce* in astrocytes and endothelial cells (Zhang et al., 2014), while pericytes would expressed only *Sgce* encoding ε-SG (Daneman et al., 2010). Together, these results combined with ours suggest that the SGC transcripts could be mainly provided by the VSMCs, the only remaining possible source in the purified brain vessels. Then, what could be the functional consequences of the *Sgcg* upregulation in Cx30^Δ/Δ^? Strong *Sgcg* overexpression in striated muscles has been shown to result in plasma membrane destabilization and muscular dystrophy with a severity varying from no damage to lethal muscle wasting disorder depending on the level of γ-SG (Zhu et al., 2001). It also led to the upregulation of *Sgca* and *Sgcb* encoding α-SG and β-SG (Zhu et al., 2001). Here, *Sgca* and *Sgcb* expression levels were not modified in Cx30^Δ/Δ^ and BBB ultrastructure and permeability were not altered, suggesting that the level of *Sgcg* expression was not sufficient to produce the same SGC alteration. It may however modify DAPCs-Ca^2+^ signaling, membrane contractility and stabilization, affecting thus the neurovascular coupling.

In summary, our study demonstrates that absence of Cx30, an astroglial gap junction protein highly expressed at the vascular interface, does not modify BBB integrity and ultrastructure, but induces the upregulation of *Sgcg* encoding γ-SG, a member of SGC which composes the muscular DAPC connecting cytoskeleton and the extracellular matrix. Furthermore, we demonstrate that all known SG units are expressed in the brain vessels suggesting the existence of a cerebrovascular SGC. Further studies will have now to determine the precise subcellular location of this cerebrovascular SGC as well as to address the physiological consequences of the upregulation of γ-SG in Cx30^Δ/Δ^ mice.

## Conflict of interest statement

The authors declare that the research was conducted in the absence of any commercial or financial relationships that could be construed as a potential conflict of interest.

## Footnotes

^1^http://www.ncbi.nlm.nih.gov/geo/query/acc.cgi?token=qtyrsakstzerxmv&acc=GSE59148 to see the data.

## References

[B1] Abbott N. J. Rönnbäck L. Hansson E. (2006). Astrocyte-endothelial interactions at the blood-brain barrier. Nat. Rev. Neurosci. 7, 41–53. 10.1038/nrn1824 16371949

[B2] Alvarez J. I. Dodelet-Devillers A. Kebir H. Ifergan I. Fabre P. J. Terouz S.. (2011). The Hedgehog pathway promotes blood-brain barrier integrity and CNS immune quiescence. Science 334, 1727–1731. 10.1126/science.1206936 22144466

[B3] Anastasi G. Tomasello F. Di Mauro D. Cutroneo G. Favaloro A. Conti A.. (2012). Expression of sarcoglycans in the human cerebral cortex: an immunohistochemical and molecular study. Cells Tissues Organs 196, 470–480. 10.1159/000336842 22738885

[B4] Anders S. Huber W. (2010). Differential expression analysis for sequence count data. Genome Biol. 11:R106. 10.1186/gb-2010-11-10-r106 20979621 PMC3218662

[B5] Argaw A. T. Asp L. Zhang J. Navrazhina K. Pham T. Mariani J. N.. (2012). Astrocyte-derived VEGF-A drives blood-brain barrier disruption in CNS inflammatory disease. J. Clin. Invest. 122, 2454–2468. 10.1172/JCI60842 22653056 PMC3386814

[B6] Bélanger M. Allaman I. Magistretti P. J. (2011). Brain energy metabolism: focus on astrocyte-neuron metabolic cooperation. Cell Metab. 14, 724–738. 10.1016/j.cmet.2011.08.016 22152301

[B7] Benfenati V. Caprini M. Dovizio M. Mylonakou M. N. Ferroni S. Ottersen O. P.. (2011). An aquaporin-4/transient receptor potential vanilloid 4 (AQP4/TRPV4) complex is essential for cell-volume control in astrocytes. Proc. Natl. Acad. Sci. U S A 108, 2563–2568. 10.1073/pnas.1012867108 21262839 PMC3038710

[B8] Boulay A. C. del Castillo F. J. Giraudet F. Hamard G. Giaume C. Petit C.. (2013). Hearing is normal without connexin30. J. Neurosci. 33, 430–434. 10.1523/JNEUROSCI.4240-12.2013 23303923 PMC6704917

[B9] Chever O. Lee C. Y. Rouach N. (2014a). Astroglial connexin43 hemichannels tune Basal excitatory synaptic transmission. J. Neurosci. 34, 11228–11232. 10.1523/JNEUROSCI.0015-14.2014 25143604 PMC6615508

[B10] Chever O. Pannasch U. Ezan P. Rouach N. (2014b). Astroglial connexin 43 sustains glutamatergic synaptic efficacy. Philos. Trans. R. Soc. Lond. B Biol. Sci. 369:20130596. 10.1098/rstb.2013.0596 25225090 PMC4173282

[B11] Constantin B. (2014). Dystrophin complex functions as a scaffold for signalling proteins. Biochim. Biophys. Acta 1838, 635–642. 10.1016/j.bbamem.2013.08.023 24021238

[B12] Dagenais C. Rousselle C. Pollack G. M. Scherrmann J. M. (2000). Development of an in situ mouse brain perfusion model and its application to mdr1a P-glycoprotein-deficient mice. J. Cereb. Blood Flow Metab. 20, 381–386. 10.1097/00004647-200002000-00020 10698076

[B13] Dallérac G. Chever O. Rouach N. (2013). How do astrocytes shape synaptic transmission? Insights from electrophysiology. Front. Cell. Neurosci. 7:159. 10.3389/fncel.2013.00159 24101894 PMC3787198

[B14] Daneman R. Zhou L. Agalliu D. Cahoy J. D. Kaushal A. Barres B. A. (2010). The mouse blood-brain barrier transcriptome: a new resource for understanding the development and function of brain endothelial cells. PLoS One 5:e13741. 10.1371/journal.pone.0013741 21060791 PMC2966423

[B15] del Zoppo G. J. Milner R. (2006). Integrin-matrix interactions in the cerebral microvasculature. Arterioscler. Thromb. Vasc. Biol. 26, 1966–1975. 10.1161/01.atv.0000232525.65682.a2 16778120

[B16] Elias L. A. Turmaine M. Parnavelas J. G. Kriegstein A. R. (2010). Connexin 43 mediates the tangential to radial migratory switch in ventrally derived cortical interneurons. J. Neurosci. 30, 7072–7077. 10.1523/JNEUROSCI.5728-09.2010 20484649 PMC2883285

[B17] Elias L. A. Wang D. D. Kriegstein A. R. (2007). Gap junction adhesion is necessary for radial migration in the neocortex. Nature 448, 901–907. 10.1038/nature06063 17713529

[B18] Ezan P. André P. Cisternino S. Saubaméa B. Boulay A. C. Doutremer S.. (2012). Deletion of astroglial connexins weakens the blood-brain barrier. J. Cereb. Blood Flow Metab. 32, 1457–1467. 10.1038/jcbfm.2012.45 22472609 PMC3421093

[B19] Goldstein J. A. Bogdanovich S. Beiriger A. Wren L. M. Rossi A. E. Gao Q. Q.. (2014). Excess SMAD signaling contributes to heart and muscle dysfunction in muscular dystrophy. Hum. Mol. Genet. 23, 6722–6731. 10.1093/hmg/ddu390 25070948 PMC4303797

[B20] Haenggi T. Fritschy J. M. (2006). Role of dystrophin and utrophin for assembly and function of the dystrophin glycoprotein complex in non-muscle tissue. Cell. Mol. Life Sci. 63, 1614–1631. 10.1007/s00018-005-5461-0 16710609 PMC11136313

[B50] Iacobas S. Iacobas D. A. Spray D. C. Scemes E. (2012). The connexin43-dependent transcriptome during brain development: importance of genetic background. Brain Res. 1487, 131–139. 10.1016/j.brainres.2012.05.062 22771707 PMC3501561

[B21] Jensen C. J. Massie A. De Keyser J. (2013). Immune players in the CNS: the astrocyte. J. Neuroimmune Pharmacol. 8, 824–839. 10.1007/s11481-013-9480-6 23821340

[B22] Jourdren L. Bernard M. Dillies M. A. Le Crom S. (2012). Eoulsan: a cloud computing-based framework facilitating high throughput sequencing analyses. Bioinformatics 28, 1542–1543. 10.1093/bioinformatics/bts165 22492314

[B23] Kunze A. Congreso M. R. Hartmann C. Wallraff-Beck A. Hüttmann K. Bedner P.. (2009). Connexin expression by radial glia-like cells is required for neurogenesis in the adult dentate gyrus. Proc. Natl. Acad. Sci. U S A 106, 11336–11341. 10.1073/pnas.0813160106 19549869 PMC2700144

[B24] Langmead B. Trapnell C. Pop M. Salzberg S. L. (2009). Ultrafast and memory-efficient alignment of short DNA sequences to the human genome. Genome Biol. 10:R25. 10.1186/gb-2009-10-3-r25 19261174 PMC2690996

[B25] Lutz S. E. Zhao Y. Gulinello M. Lee S. C. Raine C. S. Brosnan C. F. (2009). Deletion of astrocyte connexins 43 and 30 leads to a dysmyelinating phenotype and hippocampal CA1 vacuolation. J. Neurosci. 29, 7743–7752. 10.1523/JNEUROSCI.0341-09.2009 19535586 PMC2737812

[B26] Mathiisen T. M. Lehre K. P. Danbolt N. C. Ottersen O. P. (2010). The perivascular astroglial sheath provides a complete covering of the brain microvessels: an electron microscopic 3D reconstruction. Glia 58, 1094–1103. 10.1002/glia.20990 20468051

[B27] May D. Tress O. Seifert G. Willecke K. (2013). Connexin47 protein phosphorylation and stability in oligodendrocytes depend on expression of Connexin43 protein in astrocytes. J. Neurosci. 33, 7985–7996. 10.1523/JNEUROSCI.5874-12.2013 23637189 PMC6618970

[B28] North A. J. Galazkiewicz B. Byers T. J. Glenney J. R. Jr. Small J. V. (1993). Complementary distributions of vinculin and dystrophin define two distinct sarcolemma domains in smooth muscle. J. Cell Biol. 120, 1159–1167. 10.1083/jcb.120.5.1159 8436588 PMC2119721

[B29] Ozawa E. Mizuno Y. Hagiwara Y. Sasaoka T. Yoshida M. (2005). Molecular and cell biology of the sarcoglycan complex. Muscle Nerve 32, 563–576. 10.1002/mus.20349 15937871

[B30] Pannasch U. Freche D. Dallérac G. Ghézali G. Escartin C. Ezan P.. (2014). Connexin 30 sets synaptic strength by controlling astroglial synapse invasion. Nat. Neurosci. 17, 549–558. 10.1038/nn.3662 24584052

[B31] Petzold G. C. Murthy V. N. (2011). Role of astrocytes in neurovascular coupling. Neuron 71, 782–797. 10.1016/j.neuron.2011.08.009 21903073

[B32] Qu C. Gardner P. Schrijver I. (2009). The role of the cytoskeleton in the formation of gap junctions by Connexin 30. Exp. Cell Res. 315, 1683–1692. 10.1016/j.yexcr.2009.03.001 19285977

[B33] Rouach N. Koulakoff A. Abudara V. Willecke K. Giaume C. (2008). Astroglial metabolic networks sustain hippocampal synaptic transmission. Science 322, 1551–1555. 10.1126/science.1164022 19056987

[B34] Sáez J. C. Leybaert L. (2014). Hunting for connexin hemichannels. FEBS Lett. 588, 1205–1211. 10.1016/j.febslet.2014.03.004 24631534

[B35] Scemes E. (2008). Modulation of astrocyte P2Y1 receptors by the carboxyl terminal domain of the gap junction protein Cx43. Glia 56, 145–153. 10.1002/glia.20598 17990308 PMC2636557

[B36] Schütz M. Scimemi P. Majumder P. De Siati R. D. Crispino G. Rodriguez L.. (2010). The human deafness-associated connexin 30 T5M mutation causes mild hearing loss and reduces biochemical coupling among cochlear non-sensory cells in knock-in mice. Hum. Mol. Genet. 19, 4759–4773. 10.1093/hmg/ddq402 20858605 PMC2989887

[B37] Simard M. Arcuino G. Takano T. Liu Q. S. Nedergaard M. (2003). Signaling at the gliovascular interface. J. Neurosci. 23, 9254–9262. 14534260 10.1523/JNEUROSCI.23-27-09254.2003 PMC6740832

[B38] Takasato Y. Rapoport S. I. Smith Q. R. (1984). An in situ brain perfusion technique to study cerebrovascular transport in the rat. Am. J. Physiol. 247, H484–H493. 6476141 10.1152/ajpheart.1984.247.3.H484

[B39] Theis M. Jauch R. Zhuo L. Speidel D. Wallraff A. Döring B.. (2003). Accelerated hippocampal spreading depression and enhanced locomotory activity in mice with astrocyte-directed inactivation of connexin43. J. Neurosci. 23, 766–776. 12574405 10.1523/JNEUROSCI.23-03-00766.2003 PMC6741919

[B40] Thrane A. S. Rappold P. M. Fujita T. Torres A. Bekar L. K. Takano T.. (2011). Critical role of aquaporin-4 (AQP4) in astrocytic Ca2+ signaling events elicited by cerebral edema. Proc. Natl. Acad. Sci. U S A 108, 846–851. 10.1073/pnas.1015217108 21187412 PMC3021020

[B41] Urich E. Lazic S. E. Molnos J. Wells I. Freskgård P. O. (2012). Transcriptional profiling of human brain endothelial cells reveals key properties crucial for predictive in vitro blood-brain barrier models. PLoS One 7:e38149. 10.1371/journal.pone.0038149 22675443 PMC3364980

[B42] Waite A. Brown S. C. Blake D. J. (2012). The dystrophin-glycoprotein complex in brain development and disease. Trends Neurosci. 35, 487–496. 10.1016/j.tins.2012.04.004 22626542

[B43] Wolburg H. Noell S. Mack A. Wolburg-Buchholz K. Fallier-Becker P. (2009). Brain endothelial cells and the glio-vascular complex. Cell Tissue Res. 335, 75–96. 10.1007/s00441-008-0658-9 18633647

[B44] Yousif L. F. Di Russo J. Sorokin L. (2013). Laminin isoforms in endothelial and perivascular basement membranes. Cell Adh. Migr. 7, 101–110. 10.4161/cam.22680 23263631 PMC3544773

[B45] Yousif S. Marie-Claire C. Roux F. Scherrmann J. M. Declèves X. (2007). Expression of drug transporters at the blood-brain barrier using an optimized isolated rat brain microvessel strategy. Brain Res. 1134, 1–11. 10.1016/j.brainres.2006.11.089 17196184

[B46] Zaccaria M. L. Di Tommaso F. Brancaccio A. Paggi P. Petrucci T. C. (2001). Dystroglycan distribution in adult mouse brain: a light and electron microscopy study. Neuroscience 104, 311–324. 10.1016/s0306-4522(01)00092-6 11377836

[B47] Zhang Y. Chen K. Sloan S. A. Bennett M. L. Scholze A. R. O’Keeffe S.. (2014). An RNA-sequencing transcriptome and splicing database of glia, neurons and vascular cells of the cerebral cortex. J. Neurosci. 34, 11929–11947. 10.1523/JNEUROSCI.1860-14.2014 25186741 PMC4152602

[B48] Zhou J. Z. Jiang J. X. (2014). Gap junction and hemichannel-independent actions of connexins on cell and tissue functions–an update. FEBS Lett. 588, 1186–1192. 10.1016/j.febslet.2014.01.001 24434539 PMC4122521

[B49] Zhu X. Hadhazy M. Groh M. E. Wheeler M. T. Wollmann R. McNally E. M. (2001). Overexpression of gamma-sarcoglycan induces severe muscular dystrophy. Implications for the regulation of Sarcoglycan assembly. J. Biol. Chem. 276, 21785–21790. 10.1074/jbc.m101877200 11287429

